# Comments on ‘*Obstructive sleep apnea syndrome exacerbates NASH progression via selective autophagy-mediated Eepd1 degradation*’

**DOI:** 10.1093/jmcb/mjae043

**Published:** 2024-10-18

**Authors:** Jie Xiong, Suzhen Chen, Junli Liu

**Affiliations:** Shanghai Diabetes Institute, Department of Endocrinology and Metabolism, Shanghai Sixth People's Hospital Affiliated to Shanghai Jiao Tong University School of Medicine, Shanghai 200233, China; Department of Cardiovascular Medicine, First Affiliated Hospital of Nanchang University, Nanchang 330006, China; Shanghai Diabetes Institute, Department of Endocrinology and Metabolism, Shanghai Sixth People's Hospital Affiliated to Shanghai Jiao Tong University School of Medicine, Shanghai 200233, China; Shanghai Diabetes Institute, Department of Endocrinology and Metabolism, Shanghai Sixth People's Hospital Affiliated to Shanghai Jiao Tong University School of Medicine, Shanghai 200233, China

Non-alcoholic steatohepatitis (NASH) is a serious chronic disease closely associated with metabolic risk factors such as obesity, dyslipidemia, hypertension, and diabetes (Samuel and Shulman, 2018). The pathological and physiological mechanisms of NASH have not been fully elucidated, and the effective treatment for NASH is limited in clinical practice. Obstructive sleep apnea syndrome (OSAS) is a common sleep-disordered breathing disorder. A large number of clinical studies have shown that OSAS is closely related to non-alcoholic fatty liver and NASH (Aron-Wisnewsky et al., 2012). Research has shown that chronic intermittent hypoxia (CIH), a defining characteristic of OSAS, can aggravate the progression of NASH in both obese patients and obese mouse models (Savransky et al., 2007a; Aron-Wisnewsky et al., 2012). However, when constructing a CIH mouse model, intermittent hypoxia leads to decreased appetite, reduced food intake, and weight loss in mice (Savransky et al., 2007b), which is contrary to the increased appetite in OSAS patients (Mazza et al., 2020) and the general obesity seen in OSAS patients in clinical studies. This discrepancy has led to serious interference in the investigation of the pathological mechanisms of NASH in existing CIH mouse models, and the specific molecular mechanisms through which OSAS affects the development of NASH are still unclear.

Recently, to mitigate the interference encountered in the conventional CIH mouse modeling approach, we performed a pair-feeding experiment with the diabetes-induced obese mouse (Xiong et al., 2024). We found that CIH activates selective autophagy in hepatocytes by upregulating Hif1α expression, which subsequently accelerates the autophagic degradation of the DNA repair enzyme Eepd1. This process exacerbates DNA damage in hepatocytes and, consequently, promotes NASH progression. Moreover, leveraging high-throughput screening, we have identified the small molecule retigabine as an effective agent that mitigates CIH-mediated NASH progression by inhibiting the degradation of Eepd1 (Figure 1).

**Figure 1:**
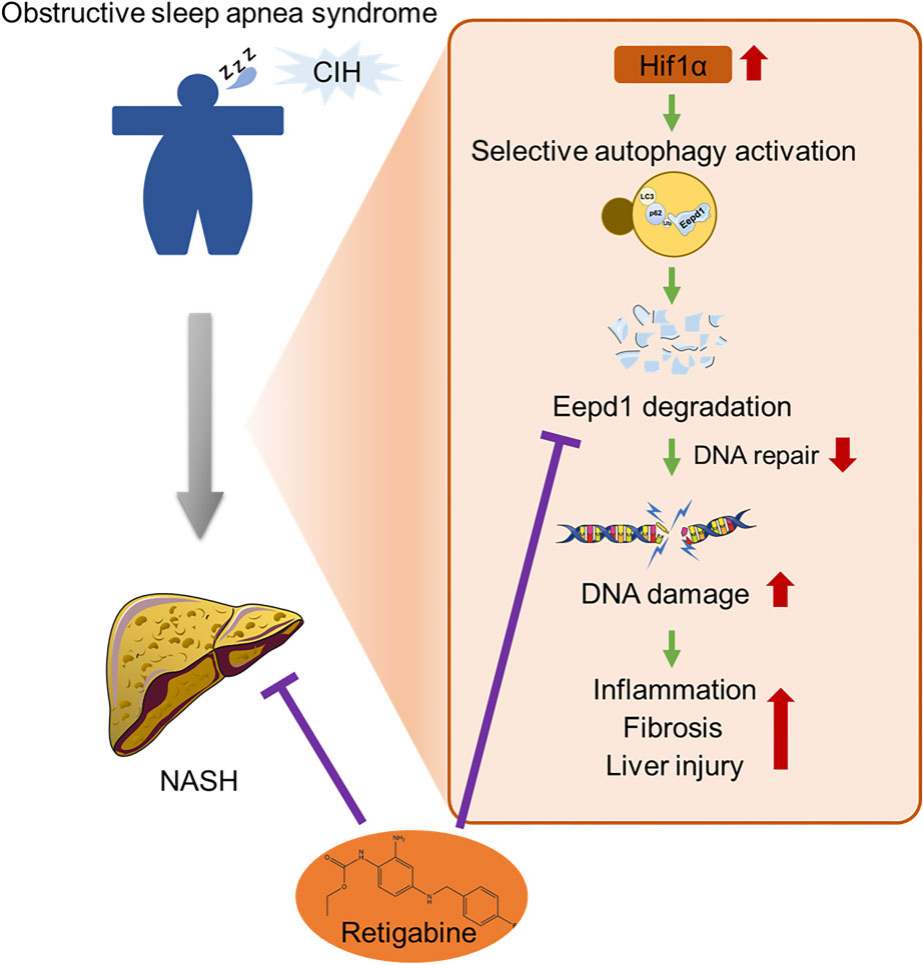
Graphical summary of the study. CIH triggers the selective autophagy in hepatocytes by upregulating Hif1α expression. This, in turn, accelerates the autophagic degradation of the DNA repair enzyme Eepd1, thereby exacerbating DNA damage in hepatocytes. Increased DNA damage aggravates liver inflammation, fibrosis, and NASH progression. Retigabine can effectively improve CIH-induced NASH by targeting CIH-mediated Eepd1 degradation. This working model was modified from Xiong et al. (2024).

DNA damage can accelerate the progression of NASH through various mechanisms, including oxidative stress (Seki et al., 2002) and induced senescence (Papatheodoridi et al., 2020). Given the exacerbation of DNA damage by CIH, we further conducted a genome-wide association analysis of the available genome-wide association study data and found a significant correlation between a single nucleotide polymorphism within Eepd1 and both metabolic traits and hypoxia-related traits, supporting the involvement of Eepd1 in CIH-induced NASH. Autophagy, a cellular process responsible for degrading damaged or unnecessary components, exerts both beneficial and detrimental effects on health and diseases (Shintani and Klionsky, 2004). Our study highlights the detrimental aspect of autophagy, as CIH stimulates the selective autophagy in hepatocytes, leading to the degradation of Eepd1, exacerbation of DNA damage, and ultimately the acceleration of NASH progression.

Due to the increasing incidence of NASH among OSAS patients, the promotion of CIH for NASH may be an important reference for future drug development. Retigabine is a potassium channel opening agent, which is clinically used in the adjunctive treatment of partial seizures in adults, and has strong anticonvulsant activity (Stafstrom et al., 2011). Our study underscores the additional potential of retigabine in ameliorating the progression of CIH-induced NASH.

In summary, our recent research has elucidated a novel mechanism underlying the exacerbation of NASH progression by CIH. Furthermore, our findings suggest that interventions aimed at mitigating CIH and restoring Eepd1 expression could serve as a promising adjunctive therapy for OSAS-exacerbated NASH. The employment of the small-molecule drug retigabine presents a fresh perspective for clinical treatment strategies targeting NASH.

*[This work was supported by grants from the Major Program of the National Natural Science Foundation of China (92357303), the National Key R&D Program of China (2021YFA0804800), the Key Program of the National Natural Science Foundation of China*

*(82330026), Shanghai Sixth People's Hospital (ynjq202102), and the Innovative Research Team of High-level Local Universities in Shanghai (SHSMU-ZDCX20212501) to J.L. This work was also supported by grants from the National Natural Science Foundation of China (82170863 and 32471208), Shanghai Rising-Star Program (21QA1407000), Lingang Laboratory (LG-QS-202205-06), Shanghai Sixth People's Hospital(ynyq202103), and Shanghai Double Hundred Program (SBR2022009) to S.C.]*

## References

[bib1] Aron-Wisnewsky J., Minville C., Tordjman J. et al. (2012). Chronic intermittent hypoxia is a major trigger for non-alcoholic fatty liver disease in morbid obese. J. Hepatol. 56, 225–233. 21703181 10.1016/j.jhep.2011.04.022

[bib2] Mazza M., Lapenta L., Losurdo A. et al. (2020). Polysomnographic and psychometric correlates of napping in primary insomnia patients. Nord. J. Psychiatry 74, 244–250. 31790624 10.1080/08039488.2019.1695285

[bib3] Papatheodoridi A.M., Chrysavgis L., Koutsilieris M. et al. (2020). The role of senescence in the development of nonalcoholic fatty liver disease and progression to nonalcoholic steatohepatitis. Hepatology 71, 363–374. 31230380 10.1002/hep.30834

[bib4] Samuel V.T., Shulman G.I. (2018). Nonalcoholic fatty liver disease as a nexus of metabolic and hepatic diseases. Cell Metab. 27, 22–41. 28867301 10.1016/j.cmet.2017.08.002 PMC5762395

[bib5] Savransky V., Bevans S., Nanayakkara A. et al. (2007a). Chronic intermittent hypoxia causes hepatitis in a mouse model of diet-induced fatty liver. Am. J. Physiol. Gastroint. Liver Physiol. 293, G871–G877. 10.1152/ajpgi.00145.2007 17690174

[bib6] Savransky V., Nanayakkara A., Vivero A. et al. (2007b). Chronic intermittent hypoxia predisposes to liver injury. Hepatology 45, 1007–1013. 17393512 10.1002/hep.21593

[bib7] Seki S., Kitada T., Yamada T. et al. (2002). In situ detection of lipid peroxidation and oxidative DNA damage in non-alcoholic fatty liver diseases. J. Hepatol. 37, 56–62. 12076862 10.1016/s0168-8278(02)00073-9

[bib8] Shintani T., Klionsky D.J. (2004). Autophagy in health and disease: a double-edged sword. Science 306, 990–995. 15528435 10.1126/science.1099993 PMC1705980

[bib9] Stafstrom C.E., Grippon S., Kirkpatrick P. (2011). Ezogabine (retigabine). Nat. Rev. Drug Discov. 10, 729–730. 21959281 10.1038/nrd3561

[bib10] Xiong J., Xu Y., Wang N. et al. (2024). Obstructive sleep apnea syndrome exacerbates NASH progression via selective autophagy-mediated Eepd1 degradation. Adv. Sci. 11, e2405955. 10.1002/advs.202405955 PMC11425227 38924647

